# Case report: Right ventricular outflow tract obstruction caused by multicomponent mesenchymal tumor

**DOI:** 10.3389/fcvm.2022.988271

**Published:** 2022-09-13

**Authors:** Shengyuan Huang, Shiye Wang, Zhixiang Tang, Yinghuan Dai, Long Song

**Affiliations:** ^1^Department of Cardiovascular Surgery, The Second Xiangya Hospital, Central South University, Changsha, China; ^2^Clinical Nursing Teaching and Research Section, Operation Room, The Second Xiangya Hospital, Central South University, Changsha, China; ^3^Department of Cardiovascular Surgery, The Second Hospital of Hebei Medical University, Shijiazhuang, China; ^4^Department of Pathology, The Second Xiangya Hospital, Central South University, Changsha, China

**Keywords:** case report, right ventricular outflow tract obstruction (RVOTO), cardiac tumor, histopathology, hemangioma

## Abstract

Right ventricular outflow tract obstruction (RVOTO) is a cause of hemodynamic instability that can lead to right ventricular dysfunction. Cardiac tumors located in the right ventricle or surrounding structures can cause RVOTO. Herein, we present a rare case of a 21-year-old male with palpitations due to RVOTO caused by a cardiac multicomponent mesenchymal tumor. The tumor was localized in the right ventricular outflow tract, resulting in right side heart enlargement, tricuspid regurgitation, and RVOTO. Hence, tumor resection was performed. The patient was in a stable condition and discharged home on the 6th post-operative day. However, histopathological examination of the tumor specimen suggested a three-component mesenchymal tumor containing mucinous components, formed blood vessels, and fibrous tissue, which is like an atypical capillary hemangioma. After seven years of follow-up, the patient had no right heart enlargement, tricuspid regurgitation, and tumor recurrence. We believe surgical treatment is effective, and this case will provide a reference for clinicians to treat and evaluate the prognosis of similar three-component mesenchymal cardiac tumor cases in the future.

## Introduction

The large autopsy series revealed that the incidence of primary cardiac tumors was 0.02%, of which 75% were benign, 25% were malignant, and myxoma accounted for 50–70% (1). But the incidence of tumor metastasis to the heart is much higher than that of the primary tumor, and researchers found that 10% of patients with tumors had cardiac metastases in an autopsy study (1). Cardiac tumors can alter hemodynamics, and when the tumor localizes in the right ventricle or surrounding structures, it may cause obstruction of the right ventricular outflow tract (RVOTO). In this case, the maximum systolic pressure difference between the right ventricle and the pulmonary artery is greater than 25 mmHg (2), and there can be many dangerous pathophysiological changes, such as right side heart enlargement, cardiac dysfunction, arrhythmias, and embolisms. Extracardiac tumor metastasis accounts for 13.7% of the total causes of RVOTO, and myxoma accounts for 6.9%, the two most common tumor-related causes (3). However, other tumor types that cause RVOTO are extremely rare. Here we report a RVOTO caused by a rare cardiac three-component mesenchymal tumor in a patient with right side heart enlargement and tricuspid regurgitation.

## Case presentation

A 21-year-old man was admitted to our hospital with a 6-day history of palpitations without chest pain, fever, and headache. The patient had a 4 years smoking history with an average of 12 cigarettes per day. There were no other significant findings in medical history or family history. Physical examination showed that body temperature was 36.1°C, pulse was 81 bpm, blood pressure was 125/75 mmHg at resting conditions, and the cardiac border was slightly enlarged on percussion. A grade 3/6 systolic murmur was heard in the third left intercostal space. Except for elevated total bilirubin (29.8 μmol/L, reference value: 5.1–17.1μmol/L) and direct bilirubin (7.5 μmol/L, reference value: 1–6.0 μmol/L), other laboratory tests revealed no specific abnormalities.

**Figure 1:**
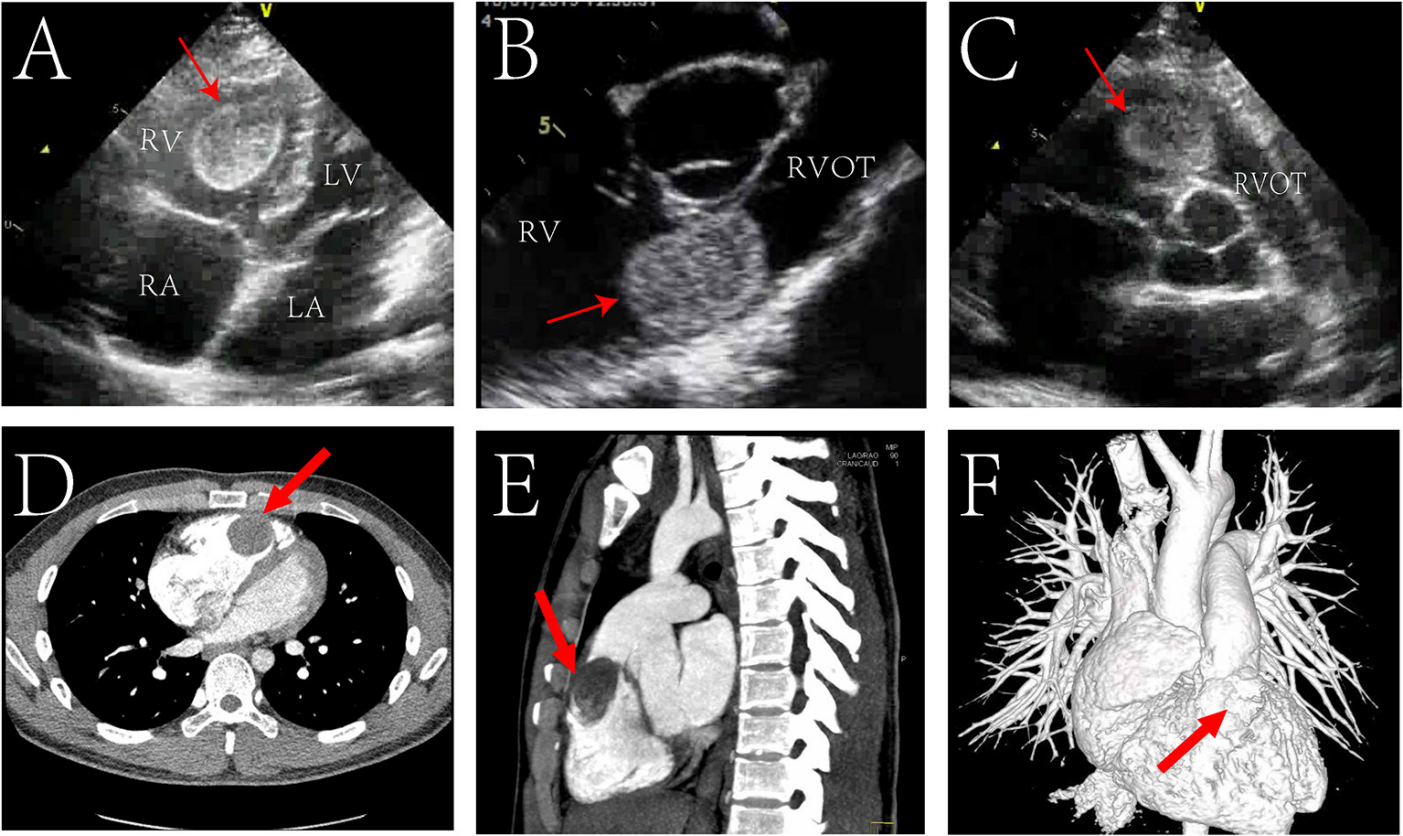
Echocardiogram pre-operatively showing a mass (red arrow) located in the in the right ventricular outflow tract (RVOT) in transthoracic four chamber cross section **(A)**, transthoracic short axis section of aorta **(B)**, transesophageal short axis section of aorta **(C)**. Preoperative cardiac Computed Tomography (CT) showed a mass (red arrow) in the RVOT in transverse view **(D)**, sagittal view **(E)**; CT angiography (CTA) showed a filling defect (red arrow) in the right ventricular outflow tract **(F)**. RV, right ventricle; LV, left ventricle; RA, right atrium; LA, left atrium.

Electrocardiogram suggested sinus rhythm with sharpened T waves. No notable abnormalities were found following chest X-ray examination. Echocardiography showed enlargement of the right atrium (50 mm), right ventricle (46 mm), and right ventricular outflow tract (31 mm) in diameter. Left atrium, left ventricle, and aorta were normal in diameter with a left ventricular ejection fraction of 74%. However, a hyperechoic space occupying mass with clear boundary (approximately 36 × 30 mm in size) was found in the right ventricle near the outflow tract (Figures 1A–C). The mass was connected to the anterior wall of the right ventricle by a pedicle and oscillated with the cardiac cycle. The tricuspid valve opened well but could not close properly, and Color Doppler Flow Imaging (CDFI) showed regurgitation under the tricuspid valve. Affected by the mass, the blood flow in the right ventricular outflow tract accelerated to 3.8 m/s, and the pressure difference (PG) across the pulmonary valve increased to 58 mmHg. Computed tomography angiography (CTA) also showed that the right atrium and right ventricle were enlarged, and a round filling defect of about 3.5^*^4.2^*^2.2 cm was seen in the right ventricular outflow tract (Figures 1D–F). Its density was roughly equivalent to that of the myocardium with clear boundaries.

Based on the above data, we diagnosed the patient with right ventricular tumor, RVOTO, and tricuspid regurgitation. To relieve the patient's obstruction and prevent potentially dangerous complications, we decided to perform surgery after fully assessing the patient's condition.

**Figure 2:**
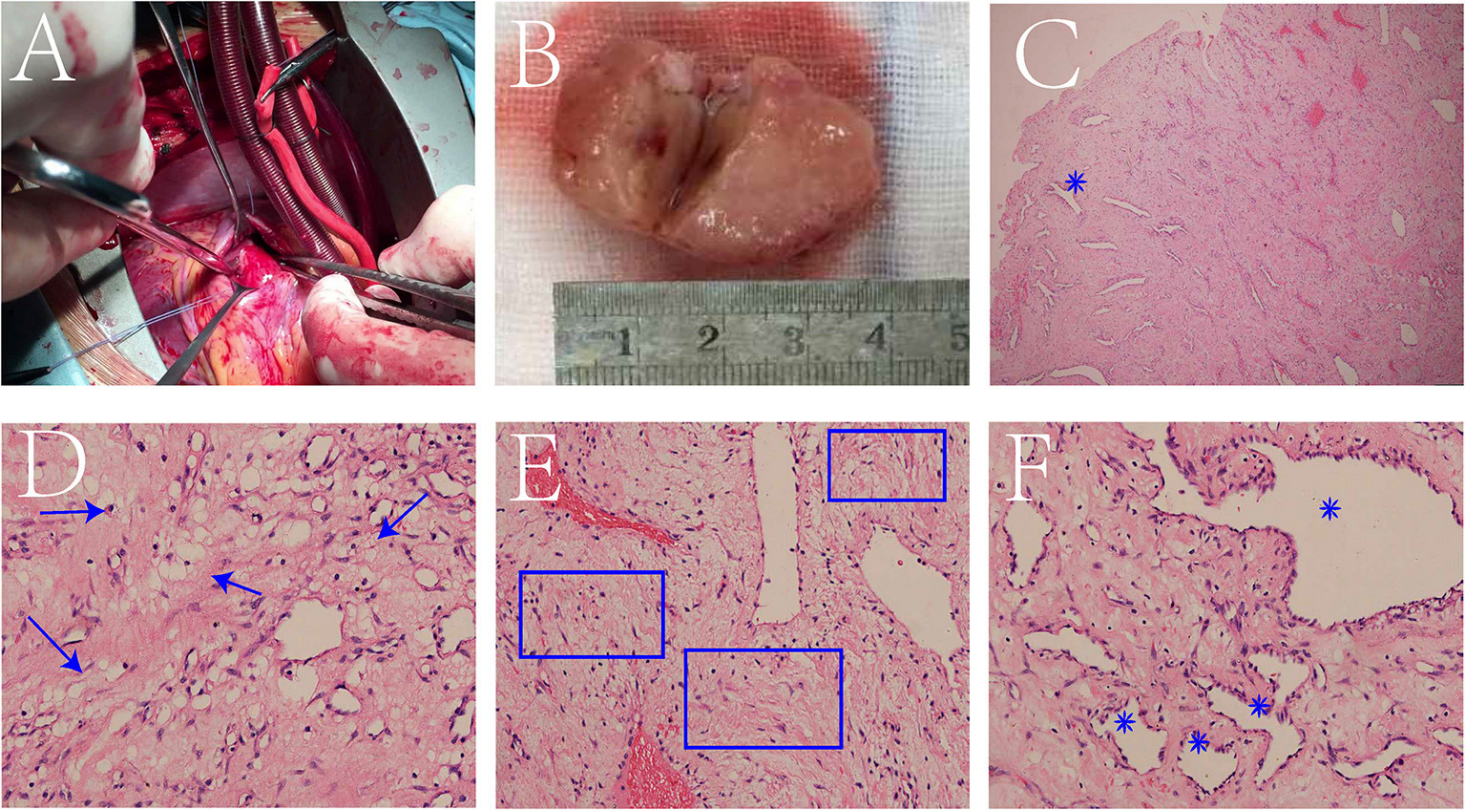
A tumor was found in the anterior wall of the right ventricle **(A)**. The specimen is a pale-yellow pedunculated ball with a texture close to myocardial tissue, and the cut surface with the “fish flesh” like texture **(B)**. Low magnification of the hematoxylin-eosin (HE) stained section showed numerous blood vessels formed (blue star) against a background of fibrous tissue **(C)**. At high magnification, mucus components (blue arrow) **(D)**, fibrous components (blue box) **(E)** and formed blood vessels (blue star) **(F)** were observed.

The surgical team performed right ventricular tumor resection. A standard median sternotomy incision was made. After pericardiotomy, the right atrium and right ventricle were found to be enlarged. After systemic heparinization, cardiopulmonary bypass was established. The surgery was performed on beating heart without aortic cross clamping. A right ventricular outflow tract incision was made, and a pedunculated tumor was found in the anterior wall of the right ventricle, with a diameter of about 3.5^*^2.5 cm (Figure 2A). The tricuspid annulus was enlarged, with mild to moderate regurgitation. The pedunculated tumor was completely excised. The incision was then sutured, heparin neutralized with protamine, and the patient was carefully transferred to the intensive care unit.

Interestingly, histopathological examination of the surgically excised specimen suggested that it was not a common myxoma, but a three-component mesenchymal tumor containing mucus, blood vessels, and fibers. The specimen was a pale-yellow pedunculated ball with a texture close to myocardial tissue, and the cut surface had a “fish-meat” like texture (Figure 2B). Low magnification of Hematoxylin-Eosin (HE) stained section shows numerous formed blood vessels against a background of fibrous tissue (Figure 2C). At high magnification, fibrous components, formed blood vessels, and mucus components were also observed (Figures 2D–F). The results indicated that the cells were positive for F8, CD34, and CD31, but negative for Ki-67, CD99, myogenin, desmin, SMA, bcl-2, and alcian blue.

**Figure 3:**
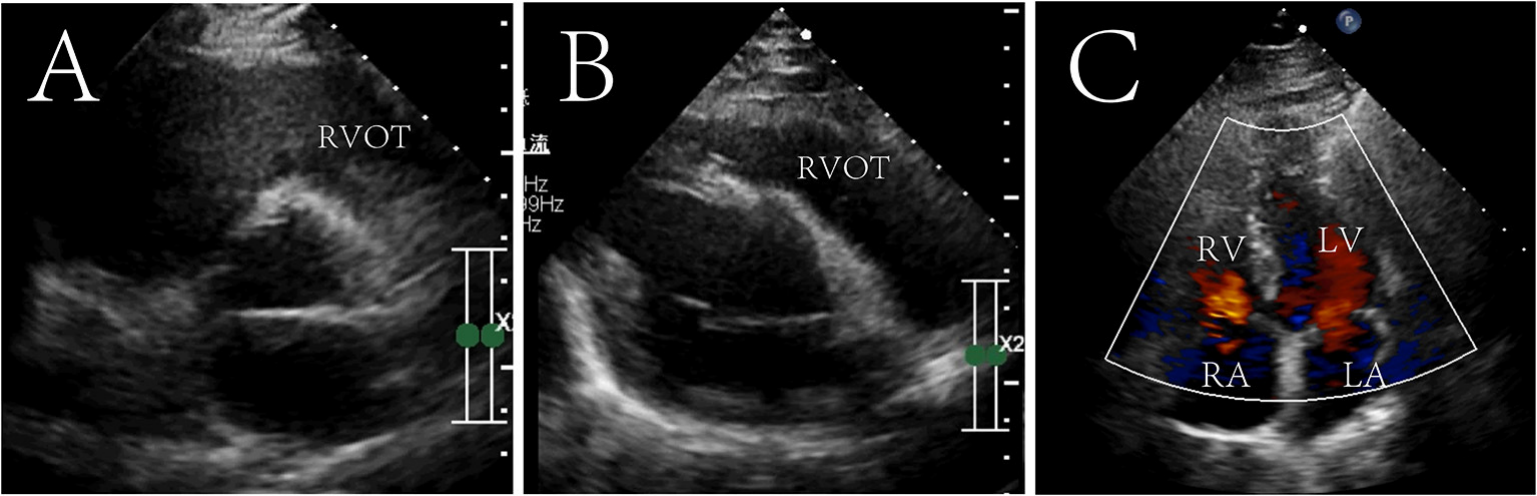
Seven-year postoperative echocardiography in transthoracic aortic short-axis views **(A)**, right ventricular outflow tract view **(B)** and four chamber cross section view **(C)**. The tumor did not recur and the right ventricular outflow tract was not obstructed. Right ventricular outflow tract, RVOT; RV, right ventricle; LV, left ventricle; RA, right atrium; LA, left atrium.

The patient recovered without palpitations and other complications and was discharged on the 6th post-operative day with a recommendation for follow-up. After 7 years of follow-up, no tumor recurrence was found on echocardiography (Figure 3 and Supplementary materials). The diameter of the right atrium decreased from 50 to 35 mm, the right ventricle from 46 to 34 mm, the RVOT from 31 to 25 mm, and the PG across the pulmonary valve to 4 mmHg. The tricuspid valve functioned normally, and no regurgitation was seen.

## Discussion

Here, we present a 21-year-old male patient with palpitations whose echocardiography revealed a hyperechoic space occupying mass in RVOT. The differential diagnosis in the presence of RVOT mass in young individuals is tumor, thrombosis, embolism, infection, foreign bodies etc. If the patient has a history of atrial fibrillation or abnormal hypercoagulability, it should be considered as thrombosis. History of fractures or prolonged immobilization after surgery in young patients can also lead to thrombosis of the lower extremities, and the thrombus may fall off into the right ventricle. If the patient has a history of long-term fever, antibiotic use, fatigue, and weight loss, the mass may be an intracardiac vegetation due to infective endocarditis. Intracardiac foreign bodies are also possible if there is a history of venous catheterization or other surgical procedures. In our case, the mass was solitary with clear boundaries, and the patient had no history of fever, atrial fibrillation, or fracture and postoperative histopathology confirmed that it was a benign cardiac tumor.

Difficulties arose in the histopathological typing of the specimens in this case. The mucinous background in HE sections can easily lead to the diagnosis of myxoma. Myxomas are typically soft and gelatinous, or hard and calcified (4). In this case, the density of the tumor tissue in the CT scan was roughly equal to that of the myocardium. The excised specimen was spheroid with a yellowish pedicle, and the cut surface had a “fish-meat” like texture. Large amount of differentiated and mature vascular components was seen in the HE section, all of which indicated that the tumor was not myxoma, but closer to the diagnosis of hemangioma.

However, this case cannot be classified as a pure hemangioma either. According to the 2015 WHO classification guidelines for cardiac and pericardial tumors, hemangioma can be classified as capillary hemangioma, cavernous hemangioma, arteriovenous malformation and intramuscular hemangioma (4). A typical HE section of a capillary hemangioma shows thin and tortuous capillaries under high magnification microscopy (5). Cavernous hemangiomas are aggregates of dilated, packed, thin-walled capillaries filled with blood cells (5, 6). The pathological images of our patient are different from the images of various types of hemangiomas in the above literature. The specimen from our case was found to have a mucinous component and many mature blood vessels in a fibrotic background under high magnification. Pathologists at our hospital spent a long time to make a diagnosis. They believed it was a multicomponent tumor of mesenchymal origin with a predominantly vascular component containing mucus and fibers. Of the four hemangiomas, it was more similar to capillary hemangioma, but had more mucous and fibrous components than typical capillary hemangiomas. Actually, there was a similar case, a family of hyperkeratotic papules reported by Dr. Peterson in 1982 (7). Histopathological examination of the biopsy showed neovascularization with mucin like change in a background of fibrosis, and a diagnosis of “myxovascular fibromas” was made. Since our patient's tumor was dominated by hemangioma components, borrowing the nomenclature “myxovascular fibromas”, we thought that this patient might be called “myxofibrous hemangioma”. However, in the existing classification system, the final histopathological diagnosis of this case was atypical capillary hemangioma with mucinous, fibrous components.

Although HE sections suggest that the tumor has a low degree of malignancy, whether it will recur is still a question, because there are no reports of an associated recurrence rate for the multicomponent mesenchymal tumor such as our case. Cardiac myxoma recurrence after surgery is about 5%, atypical cases are more likely to recur (8), and the cumulative recurrence rate is higher in younger patients (13%) (9). A review of 200 cases of cardiac hemangioma found recurrence in 3 patients and one manifested angiosarcoma at the site of hemangioma (10). A study reported that the recurrence rate of cardiac Fibroelastoma was 12.2% (11). Now the question is, do multicomponent tumors increase recurrence rates? Fortunately, our patient at 7-years follow-up showed no tumor recurrence and the heart was functioning normally.

Different tumor types and tumor location in right ventricle can cause RVOTO. We reviewed the literature on RVOTO caused by cardiac tumors reported in the past decade (Table 1). In terms of tumor types, the literature reported primary tumors such as fibroma (12), leiomyoma (13), hemangioma (17), rhabdomyoma (20), and metastatic cardiac tumor from sigmoid colon cancer (16), testicular tumor (15), neuroendocrine tumor (25) etc. Interestingly, tumors not located in the right ventricle can also cause obstruction, such as tumors located in the right atrium, interventricular septum, and anterior mediastinum (24, 32, 33).

**Table 1:** Cases of RVOTO due to cardiac tumors reported in the past decade.

**Year of publication**	**Age**	**Tumor type**	**Location**	**References**
2012	6 months	Primary fibroma	Right ventricle	(12)
2012	7 months	Primary cardiac leiomyoma	Ventricular septum	(13)
2012	70 years	Malignant fibrous histiocytoma	RVOT	(14)
2012	42 years	Testicular tumor metastasis	Right ventricle	(15)
2013	70 years	Sigmoid colon cancer metastasis	Right ventricle	(16)
2014	41 years	Cardiac capillary hemangioma	Right ventricle	(17)
2014	24 years	Primary leiomyoma	Right ventricle	(18)
2016	62 years	Renal cell carcinoma metastasis or tumor thrombus	Right ventricle	(19)
2016	6 months	Rhabdomyoma	Right ventricle	(20)
2017	5 months	Rhabdomyoma	RVOT, pulmonary valve annulus	(21)
2018	46 years	Yolk sac tumors metastasis	Right ventricle	(22)
2018	67 years	Metastatic adenocarcinoma	Right ventricle and pulmonary artery	(23)
2018	15 months	Primary fibroma	Right-sided interventricular septal	(24)
2018	68 years	Metastatic neuroendocrine tumor	Right ventricle	(25)
2019	9 days	Teratoma	Right ventricle	(26)
2020	60 years	Neuroendocrine carcinoma metastasis	Right ventricle	(27)
2020	42 years	Primary undifferentiated spindle cell sarcoma	Right ventricle, pulmonary trunk	(28)
2021	54 years	AIDS-associated primary cardiac lymphoma	Right atrium and ventricle	(29)
2021	44 years	Myxoma	Right ventricle	(30)
2021	5 years	Cardiac inflammatory myofibroblastic tumor	Pulmonary valve, extending into the main pulmonary artery	(31)

Similar case of capillary hemangioma has been reported by Shekarkhar et al. (17). Their case was a 41-year-old man who complained of chest pain and fatigue. Ultrasound showed normal size right heart, tricuspid regurgitation, and a 1.6^*^1.8 cm mass located in the RVOT at the base of the septum. They also removed the tumor, and pathological examination under the high magnification microscope found neoformed vessels filled with blood. Compared to their case, our patient did not show symptoms of chest pain and fatigue, the right heart was significantly enlarged, and the tumor was located in the anterior wall of the right ventricle. And there was no recurrence after many years of follow-up, and the tricuspid regurgitation also disappeared.

Notably, the surgical team had difficulty deciding whether to perform surgery for tricuspid regurgitation (TR) in this patient. The patient had a marked enlargement of the right atrium, dilated tricuspid annulus to 37 mm, and mild TR on Echocardiography, which were suggestive of surgical intervention. However, after consulting the European Society of Cardiology (ESC) guidelines of 2012 (34), it was found that tricuspid valve surgery is a class IIa recommendation for patients with secondary TR when annulus is larger than 40 mm. In addition, considering that the tricuspid valve was not invaded by the tumor and the left heart function was normal, we speculated that the TR would disappear after hemodynamic correction. Therefore, we did not perform tricuspid valve surgery, and 7 years later the TR disappeared which confirmed our conjecture. This suggests that when such patients have TR, if the valve annulus does not exceed 40 mm, the tumor does not invade the tricuspid valve, and the left heart function is normal, TR may disappear spontaneously without tricuspid valve surgery.

In conclusion, we report a rare case of cardiac tumor induced RVOTO in a patient with right atrial and ventricular enlargement and tricuspid regurgitation. More interestingly, histopathological examination of the tumor revealed it was a three-component mesenchymal tumor with mucinous components and formed blood vessels in a fibrotic background. In the existing WHO classification of cardiac tumors, it is more like an atypical capillary hemangioma. Resection of the tumor relieved symptoms and there was no recurrence after 7 years. We case can be a reference for clinicians to treat similar three-component mesenchymal cardiac tumor cases in the future.

## Data availability statement

The original contributions presented in the study are included in the article/Supplementary material, further inquiries can be directed to the corresponding author/s.

## Ethics statement

The studies involving human participants were reviewed and approved by Ethics Committee of the Second Xiangya Hospital of Central South University, Changsha, China. The patients/participants provided their written informed consent to participate in this study. Written informed consent was obtained from the individual(s) for the publication of any potentially identifiable images or data included in this article.

## Author contributions

SH drafted the manuscript. LS designed the study and were responsible for the collection of data or analysis. SW, ZT, and YD analyzed the data and revised the manuscript. All authors read and approved the final manuscript.

## Funding

This work was supported by the Natural Science Foundation of Hunan Province (Grant No. 2021JJ30951) and Clinical Technology Innovation Guide Project of Hunan Province (Grant No. 2020SK53421).

## Conflict of interest

The authors declare that the research was conducted in the absence of any commercial or financial relationships that could be construed as a potential conflict of interest.

## Publisher's note

All claims expressed in this article are solely those of the authors and do not necessarily represent those of their affiliated organizations, or those of the publisher, the editors and the reviewers. Any product that may be evaluated in this article, or claim that may be made by its manufacturer, is not guaranteed or endorsed by the publisher.
